# Oral post-surgical complications in patients with hemophilia and von Willebrand disease

**DOI:** 10.1016/j.htct.2025.103936

**Published:** 2025-07-05

**Authors:** Luisa Catarina Porfirio de Sousa, Elanne Cristina Garcia da Costa Félix, Wagner Hespanhol, Raquel dos Santos Pinheiro

**Affiliations:** Instituto Estadual de Hematologia Arthur Siqueira Cavalcanti (Hemorio), Rio de Janeiro, Rio de Janeiro, Brazil

**Keywords:** Oral surgery, Oral hemorrhage, Hemophilia A, Hemophilia B, von Willebrand diseases

## Abstract

**Objective:**

To determine the prevalence of post-surgical complications in patients with hemophilia and von Willebrand disease.

**Methods:**

A prospective, cross-sectional study with descriptive and exploratory data analysis was conducted at the outpatient clinic of the Arthur de Siqueira Cavalcanti State Institute of Hematology (Hemorio). The sample included 26 patients who underwent tooth extraction following the protocols of the Brazilian Ministry of Health.

**Results:**

The prevalence of post-surgical complications identified in the study was 26.07 %, with 15.38 % of cases presenting bleeding after extraction.

**Conclusion:**

The prevalence of postoperative complications found in this study was notably higher in patients with von Willebrand disease, followed by those with severe hemophilia.

## Introduction

Coagulopathies are hemorrhagic disorders caused by deficiencies in one or more coagulation factors, which may be either quantitative or qualitative.^1^ Patients with coagulopathies can clinically present with bleeding of varying severity, either spontaneous or post-traumatic, predominantly in the oral cavity.^2^

Hemophilia is an X-linked hereditary bleeding disorder, characterized by the deficiency of coagulation factors VIII (hemophilia A) or IX (hemophilia B).^1^^,^^3^^,^^4^ Its prevalence is approximately 1 per 5000–10,000 live male births for hemophilia A, and 1 per 30,000–40,000 live male births for hemophilia B.^1^^,^^4^^,^^5^ According to the consensus of the International Society of Thrombosis and Haemostasis, this dyscrasia is classified based on plasma levels of factor VIII (FVIII) or factor IX (FIX): severe (<1 % of normal), moderate (1–5 % of normal), and mild (5–40 % of normal).^1^^,^^4^

Von Willebrand disease (VWD) is a hereditary coagulopathy caused by a genetic deficiency in von Willebrand factor (VWF), a plasma glycoprotein. It is the most prevalent coagulopathy among hemorrhagic disorders, affecting approximately 1 in every 100–1000 individuals. VWD is divided into three types: Types 1 and 3 involve quantitative deficiencies of VWF, while Type 2 is associated with qualitative defects.^1^^,^^6^

Patients with coagulopathies often neglect their oral health due to a fear of bleeding during toothbrushing and flossing.^7^ Among dental procedures, oral surgeries carry the highest risk of bleeding and complications, whether intraoperative or postoperative.^1^ Dental professionals must follow proper care protocols, including conducting a thorough medical history, consulting with the patient’s hematologist to discuss the severity of the condition, and evaluating the risks of proposed procedures. Familiarity with local hemostatic measures, such as the use of fibrin sealants, proper anesthetic techniques, tranexamic acid, trichloroacetic acid, and other antifibrinolytics, is essential to minimize complications during treatment.^2^^,^^8^^,^^9^

In this context, the present study aims to identify the prevalence of post-surgical complications in patients with hemophilia and von Willebrand disease.

## Methods

This research was submitted and approved by the Research Ethics Committee of Hemorio under number CAAE 57792122.3.0000.5267, in accordance with Resolution 466/2012 of the National Health Council.

This prospective, cross-sectional research using descriptive and exploratory data analysis was conducted at the dental outpatient clinic of Hemorio from July to December 2022. The sample size of 1493 patients was calculated, taking into account the number of patients diagnosed with Hemophilia A, Hemophilia B, and Von Willebrand Disease enrolled at Hemorio in the last two years. From the sample calculation, a maximum sample size of 306 was obtained, with a 5 % margin of error and a 5 % significance level. Since patients voluntarily seek dental care, the minimum sample size was not reached. The inclusion criteria were: being over 18 years old, requiring tooth extraction, having a panoramic radiograph, and voluntarily consenting to participate by signing the informed consent form. Twenty-six participants who met the inclusion and exclusion criteria after clinical examination were enrolled.

Data collection took place in a dental office under artificial lighting, using a flat mirror. Panoramic radiographs were taken, and participants completed a questionnaire developed by the researcher, which included questions on oral hygiene habits and dental bleeding history.

On the day of extraction, participants completed a health history questionnaire, and their vital signs were measured. After the extraction, an intraoperative form was filled out to document technical details of the procedure, such as tooth impaction, odontosection, osteotomy, and crown fracture. Participants returned for a follow-up visit 7–10 days later for suture removal and clinical evaluation, at which time a postoperative assessment form was completed.

Surgical procedures followed protocols of the Brazilian Ministry of Health.^1^^,^^10^ Transfusion strategies were implemented to elevate FVIII or FIX levels to 80 % with a single preoperative dose for patients with hemophilia. Additional doses or oral tranexamic acid (25 mg/kg every eight hours) were administered in the days following the procedure, depending on the surgical details and the patient's bleeding history.

For patients with von Willebrand disease, the goal was to increase plasma levels of the deficient protein. Treatment options included desmopressin (DDAVP) or FVIII/VWF concentrates, often supplemented with oral tranexamic acid (25 mg/kg every eight hours), as recommended by a hematologist.

Local hemostatic measures included the application of a lyophilized hydrolyzed collagen hemostatic sponge (Hemospon, Maquira, Maringá, Paraná, Brazil), 3–0 silk sutures, and a hemostatic paste made by combining macerated tranexamic acid tablets with 0.2 % chlorhexidine digluconate gel.

Postoperative recommendations included maintaining regular oral hygiene, refraining from smoking and consuming alcoholic beverages, and eating soft, room temperature or cold foods for the first 48 h. Patients were advised to apply extraoral ice packs and take relative rest during the first 24 h, and rinse with a 0.12 % chlorhexidine digluconate mouthwash for one week. Moreover, they were warned to avoid exposure to sun, and refrain from vigorous rinsing, using a straw, or spitting during the first 72 h. In case of bleeding, patients were instructed to bite on a gauze pad for 15 min and return to the clinic if the bleeding persisted.

Data were tabulated using Microsoft Excel, followed by quantitative and descriptive statistical analysis.

## Results

In the studied sample, von Willebrand disease was the most prevalent coagulopathy, accounting for 46.2 % of participants, followed by Hemophilia A (38.5 %) and Hemophilia B (15.4 %). Male patients were more prevalent, representing 57.7 % of the sample. The mean age was 39.9 years (standard deviation: 15.1 years), with the most represented age groups being 21–30 years and 31–40 years, each comprising 30.8 % of the participants (Table 1).

**Table 1:** Profile of research participants.

Variable		n (%)
Gender	Female	11 (42.3)
	Male	15 (57.7)
Type of coagulopathy	von Willebrand disease	12 (46.2)
	Hemophilia A	10 (38.5)
	Hemophilia B	4 (15.4)
Age (years)	21–30	8 (30.8)
	31–40	8 (30.8)
	41–50	3 (11.5)
	51–60	3 (11.5)
	61–70	3 (11.5)
	71–80	1 (3.8)

Regarding oral hygiene habits, all participants reported brushing their teeth daily; 46.2 % brushed three times a day, 42.3 % twice a day, and 11.5 % four or more times daily. Additionally, 84.6 % reported cleaning their tongue during routine brushing. When asked about flossing, 69.2 % of participants reported flossing, with 33.3 % doing so sporadically, 33.3 % at least once a day, 11.1 % twice daily, 11.1 % three times daily, and 11.1 % four or more times daily (Table 2).

**Table 2:** Oral hygiene habits.

	Yes n (%)	No n (%)	Frequency per day n (%)				
			1	2	3	4 or more	Sporadic use
Brushing	26 (100)	0 (0)	0 (0)	11 (42.3)	12 (46.2)	3 (11.5)	0 (0)
Tongue brushing	22 (84.6)	4 (15.4)	4 (18.2)	10 (45.5)	7 (31.8)	1 (4.5)	0 (0)
Flossing	18 (69.2)	8 (30.8)	6 (33.3)	2 (11.1)	2 (11.1)	2 (11.1)	6 (33.3)

When questioned about receiving hygiene guidance, 18 participants (69.2 %) reported having received some type of orientation, primarily from a dentist (83.3 %), followed by family members (11.1 %) and others (5.6 %). All patients reported having visited a dentist at least once before.

Twenty-two participants (84.6 %) reported experiencing bleeding episodes in their lifetime, with 12 (54.5 %) requiring urgent care for these episodes. Regarding tooth extractions, 21 participants (80.8 %) had previously undergone dental extractions, with 15 (71.4 %) reporting bleeding after the procedure. None of the patients had a history of inhibitors.

The absence rate for follow-up consultations after surgery was five (19.2 %) participants. Among those who attended follow-ups, complications related to the surgical procedure were observed in 23.07 % of the cases. Bleeding was reported in 47.6 % of these cases, with 40 % (15.38 % of the total sample) requiring emergency care to manage the bleeding.

Hematomas, hospitalizations, and other postoperative complications were evaluated. None of the participants required hospitalization. One patient developed a hematoma at the extraction site, and two patients experienced complications unrelated to bleeding. All teeth associated with bleeding complications were posterior and located in the maxilla. These included three third molars (one impacted) and one premolar with extensive caries and a crown fracture, leaving only a root remnant. Additionally, two molars developed alveolitis: one in the maxilla and one in the mandible. Table 3 summarizes the postoperative complications and their details.

**Table 3:** Postoperative complications.

	Event	Diagnosis	Gender	Age	Tooth	Indication for extraction	Postoperative recommendations	Details
1	Hemorrhage	Hemophilia B - Severe	Male	31	28	Caries	Not followed	- The patient reported not having taken the recommended dose of tranexamic acid as prescribed by the physician.
2	Hemorrhage	von Willebrand disease*	Female	31	28	Orthodontic	Followed	- With a history of bleeding during previous extractions.
3	Hemorrhage	von Willebrand disease*	Male	36	28	Impaction	Followed	- Osteotomy
4	Hemorrhage	Hemophilia A - Severe	Male	56	24	Caries/Radicular remnant	Not followed	- Osteotomy/The patient reported not having taken the recommended dose of tranexamic acid as prescribed by the physician.
5	Alveolitis	von Willebrand disease*	Female	48	26	Caries/Radicular remnant	Followed	- Poor hygiene in the extraction region
6	Alveolitis	Hemophilia A - Severe	Male	34	38	Impaction	Not followed	- Osteotomy/The patient reported smoking during the postoperative period.

⁎ Without specification of type.

Table 4 presents data on the reassessments conducted for patients with postoperative complications. The first reassessment (Reassessment 1) was performed within seven days of the procedure to address urgent needs. The second reassessment (Reassessment 2) occurred within 14 days after the extraction and included urgent care as necessary. Only one of these patients did not return for the second reassessment.

**Table 4:** Postoperative reassessments.

Reassessment 1				Reassessment 2		
	Suture	Presence of malformed clot	Healing	Suture	Healing	Bleeding
1	Intact	Yes	Unsatisfactory	Intact	Satisfactory	No
2	Intact	Yes	Satisfactory	–	–	–
3	Intact	Yes	Unsatisfactory	Intact	Satisfactory	No
4	Intact	Yes	Satisfactory	Intact	Satisfactory	No
5	Intact	Yes	Unsatisfactory	Intact	Satisfactory	No
6	Intact	No	Satisfactory	Intact	Satisfactory	No

## Discussion

In the present study, a postoperative complication rate of 23.07 % was identified, with 15.38 % attributed to bleeding episodes. In comparison, Franchini et al.^11^ reported a 3.1 % bleeding rate in 288 procedures using fibrin glue for local hemostasis. Similarly, Hsieh et al.^12^ observed a bleeding incidence of 18.9 % (10 out of 53 extractions) with hemostatic measures like gelatin sponges and oxidized cellulose. Bajkin and Dougall^13^ found general bleeding rates of 11.9 % and 11.4 % for pre- and post-procedure factor concentrate use. Yagyuu et al.^14^ reported post-extraction bleeding in 16.3 % of cases (9 out of 55), while Cesconetto et al.^15^ found five cases of bleeding among 73 patients. The higher complication and bleeding rates in this study may be due to the small sample size and the inclusion of all patients undergoing tooth extractions.

Effective local hemostasis is essential to reduce bleeding risk. Commonly used materials, such as collagen sponges, fibrin sealants, and oxidized cellulose, stabilize clots, and non-absorbable sutures should be applied.^1^ Epsilon aminocaproic acid and 10 % trichloroacetic acid are also used for minor gingival bleeding.^1^^,^^16^ In this study, Hemospon filled the socket, and 3–0 silk sutures were applied. A paste of macerated tranexamic acid tablets mixed with 0.2 % chlorhexidine gel was used, chosen for its consistency and ability to concentrate the material at the extraction site. Fibrin glue and other local hemostatics were unavailable, but the protocol remained effective. The literature suggests using tranexamic acid as a mouthwash, intravenously, or mixed with local anesthetics for direct application.^1^^,^^9^^,^^16^ Due to the risk of dislodging the clot, mouthwash was not recommended after extraction, and tranexamic acid was used only in paste form.

When appropriately indicated, systemic tranexamic acid offers significant benefits, particularly in controlling bleeding and promoting healing.^9^ The study results support the effectiveness of the protocol of the Brazilian Ministry of Health with tranexamic acid, as only four patients experienced bleeding, two of which were due to underdosing.

In cases of post-surgical hemorrhage, the hematologist must be contacted for transfusion replacement, followed by local anesthesia, suture removal, socket curettage, clot removal, and management of granulation tissue. Local hemostasis should be achieved using collagen sponges, fibrin sealants, or other hemostatic materials, along with firm sutures and antifibrinolytic paste mixed with 0.2 % chlorhexidine gel.^1^ No patient in this study had recurrent bleeding after this procedure.

This study also observed two cases of alveolitis: one dry socket and one purulent socket. The dry socket patient presented bone pain, halitosis, and smoking during the postoperative period, consistent with the report by Kuśnierek et al.,^17^ who found smoking increases the risk of alveolitis. The purulent socket patient had poor oral hygiene in addition to the symptoms above. While the exact causes of alveolitis are unclear, poor hygiene is believed to be a contributing factor.^18^

Despite clear verbal and written postoperative instructions, three patients experienced complications due to neglecting these guidelines. Silva^19^ emphasized the need for healthcare providers to tailor instructions to the understanding of patients to minimize complications and improve postoperative quality of life. Thus, it is the healthcare professional’s responsibility to communicate care guidelines effectively to reduce the risk of forgetfulness and associated complications.

Good oral hygiene is crucial for patients with blood disorders, as healthy gums do not bleed spontaneously.^1^ In this study, 100 % of patients reported daily tooth brushing, 84.6 % brushed their tongues, and 69.2 % used dental floss. Czajkowska et al.^20^ found worse interdental hygiene in patients with blood disorders compared to healthy individuals, often due to a fear of gingival bleeding. Regular dental visits, at least every six months, are recommended for monitoring and prevention.

The limitations of this study include the small sample size and the unavailability of ‘gold standard’ medications like fibrin glue. However, the prospective design minimizes exposure determination bias, offering an advantage over retrospective studies. Further prospective studies are needed to optimize the safety of oral surgeries for patients with blood dyscrasias and minimize associated risks.

In conclusion, the prevalence of postoperative complications in this study was notably higher in patients with von Willebrand disease, followed by those with severe hemophilia. With this knowledge, dental surgeons can feel more confident about performing surgery on patients with blood dyscrasias.

## Conflicts of interest

The author declares no conflicts of interest.
